# Exiled at home: British Muslims’ experiences of integration

**DOI:** 10.3389/fsoc.2023.1172057

**Published:** 2023-09-22

**Authors:** Sara Elsayed

**Affiliations:** School of Sociology and Social Policy, University of Nottingham, Nottingham, United Kingdom

**Keywords:** exile, integration, home, belonging, identity, Muslims

## Abstract

This article takes up the question of Muslim integration in the United Kingdom with one main argument: British Muslims, including those who are UK-born, endure wider exilic narratives that stand in clear contradiction to a rooted sense of belonging and equal citizenship. Referring to data from 12 months of ethnographic research, this article argues that integration as a lived experience for this community is ironically characterised by a range of exilic narratives entailing stereotyping, misrecognition, misrepresentation, and inequalities that put their sense of Britian as ‘home’ at stake. While these conditions do not necessarily work in the same way for all Muslims across their differences, they generally shape—in many different ways—their perceptions and understandings of belonging, home, and integration. Exploring everyday integration practices and dynamics in a local community, this paper discusses Muslims’ sense of belonging and the barriers they encounter in achieving a sense of home. It investigates the roles of fear and citizenship built in inequalities in creating an exilic space that impinges on Muslims’ sense of belonging. The analysis at the end extends this to highlight the responses and approaches Muslims adopt in their efforts to construct belonging in an exilic context.

## Introduction

In *Reflections on Exile*, Said (2013) describes exile as a *forced* rupture between a human being and a native place. It is a state of being that implies a sense of homelessness and non-belonging. In most literature, exile is portrayed as a geographical concept referring to the ‘uprooted experience of living abroad, away from one’s motherland’ (Zeng, 2010, 1). With this perspective in mind, traditional scholarship on exile focusses on (in)voluntarily dislocated immigrant groups (outsiders) in foreign host countries (Harlem, 2010; Das et al., 2021). Exile from this perspective is typically seen as a deprivation of the homeland and its rhythms of life (Graham and Khosravi, 1997, 115). Those who suffer the exile are necessarily deprived of a homeland in geographical and spatial terms (Berg, 1996, 4). The dynamics of displacement from the home country followed by emplacement and home remaking in a new country bring with it a range of experiences such as alienation, marginality, uprootedness, uncertainty, and liminality (Graham and Khosravi, 1997; Marciniak, 2006; Pearlman, 2023). However, these experiences can also occur without physical displacement (voluntary or involuntary) and could equally be experienced in one’s homeland (Graham and Khosravi, 1997). In this paper, I argue that shades of exile can be experienced within one’s homeland and without geographical displacement (Gass, 1990; Zeng, 2010; Burr, 2014; Chouana, 2019). Thus, deprivation of the homeland is not necessarily geographical, but one that relates to a lack of belonging or, as Burr argues, to missing ‘being-at-home’ (Burr, 2014, 49). Exile, in line with this, is a state (or a place) in which one fails to connect to self, others, and place, and leads to a sense of alienation and estrangement, jeopardising possibilities for self-fulfilment, links to community, and feelings of security (Pearlman, 2023, 165). Exile is, therefore, a place and/or condition where one’s true self and intrinsic worth is lost, and where one’s entitlement to basic rights are either not respected or are unacknowledged (Müller, 2016). A home, conversely, is a place where we rather ‘could or can be ourselves, feel at ease, secure, able to express ourselves freely and fully’ (Tucker, 1994, 184).

For decades, Muslims have been at the centre of integration debates in the United Kingdom. As a problematized faith community, Muslims have largely been seen as a menace with disloyal, culturally separatist, and younger generations vulnerable to radicalisation (Home Office, 2001; Home Office 2005; Blair, 2006; Briggs et al., 2006; Kelly 2006; Straw, 2006; Cameron, 2011, 2015; Blair, 2016; Casey, 2016; Shawcross, 2023). The publication of the Shawcross review—the latest governmental review of Prevent—reveals that Muslims continue to remain at the centre of political and security attention in the United Kingdom (Shawcross, 2023). This attention is led by an apprehension that Muslims in Britain are self-segregating, ghettoised, and not leading a British way of life—issues perceived in public and political discourse as threatening to national unity and community cohesion. Yet, most academic research argues that religious Muslims show a strong inclination towards integration in the wider British society, that they cherish British culture (e.g. in terms of democracy, rule of law and equality) and that they are no different from other communities in the United Kingdom in terms of their sense of belonging to the United Kingdom (Maxwell, 2006; Pettersson, 2007; Kibria, 2008; Kundnani, 2008; Uberoi and Modood, 2009; Hopkins, 2011; O’Toole and Gale, 2013; Mustafa, 2015; Oskooii and Dana, 2018; Ahmed, 2019; Phoenix, 2019).

Based on 12 months of ethnographic fieldwork in a Muslim concentrated neighbourhood in Birmingham, this article offers a contextualised account of how British Muslims feel about their belongingness in the context of their integration experiences. The main focus of the paper, therefore, is to move beyond theoretical debates and political reflections on Muslim integration towards an in-depth understanding of what it means to (not) belong, based on systematic engagement with real-world stories and everyday experiences. It investigates the complex interplay of fear and citizenship deficits which together work instrumentally in restricting Muslims’ sense of belonging and shaping a state of exile. The conception of exile, as used here, captures the paradoxical everyday experiences of British Muslims as they, despite feeling and identifying as British, remain perceived as leading a parallel and non-British way of life. Little research has been conducted to explore exilic conditions in communities other than those typically considered as exiles (e.g. refugees and asylum seekers; Jamal and Chapman, 2000; Schneider and Crul, 2010; Cherti and McNeil, 2012; Ahmed, 2019; Pearlman, 2023) and although research to date does provide important insights into everyday integration and belonging through investigating the construction of group identities in everyday settings (Cherti and McNeil, 2012, 4–5), focussing on integration with reference to group identities has not provided a detailed understanding of how certain home contexts may shape exile-like environments that impact on self-identification and shape the relationship between the self and the other.

Following a discussion of my theoretical and methodological approaches, the article explores participants’ experiences of exilic conditions in the context of their integration. This is covered in two subsections: the first outlines the role of fear as an exilic narrative in hindering a sense of home; and the second discusses the drawbacks of a citizenship which emphasise feelings of inequality and non-recognition among Muslims. This will be followed by a discussion of the approaches Muslims take to maintain a sense of belonging and feeling ‘at home’ in an exilic context.

## Theoretical framework

Critical to a discussion of belonging, home, and exile is the notion of citizenship. Citizenship theories provide a compelling framework for understanding different citizenry entitlements that closely link to debates over integration, belonging, and inclusion. Performing its role in distinguishing between ‘insiders’ and ‘outsiders’, citizenship reaches to the heart of debates over belonging. Citizenship as a legal framework gives foundation to the institutional structure essential to help migrants and ethnic communities in general feel ‘at home’ (Penninx, 2004; Anderson and Hughes, 2015).

Underpinning successful integration are citizenship rights that define how inclusive the integration process can be. Foundational to its very concept, citizenship is built on hierarchies and is designed largely to include/exclude who can/cannot be a member of the national community. It is, as De Genova puts it, ‘an enduring framework for the production of hierarchies and exclusions’ (De Genova, 2015, 196). Citizenship cannot therefore be understood as mere legal entitlements distinguishing citizens from non-citizens and a discussion of citizenship should rather be extended to include the daily practices of citizenship that may render the legal rights and entitlements of citizenry an ‘illusory’ status. Citizenship as a formal and legal status does not necessarily guarantee inclusion (Anderson and Hughes, 2015). Exile, as perceived in this article, is a condition and a lived experience—rather than a place—produced through day-to-day reminders of the unfulfilled promise of equal citizenship and inclusion. Citizenship can be understood in more than one way and its promise of inclusivity can be realised to a greater or lesser degree. Different schools approach citizenship in different ways. While liberals focus mainly on civil and political individual rights (Marshall, 1950; Kymlicka and Norman, 1994; Rawls, 1999; Lister and Pia, 2008), communitarians focus on community rights (Walzer, 1982; Sandel, 1984; Taylor, 1992; Sandel et al., 1998; Loobuyck, 2016), and cultural pluralists focus on cultural particularistic rights (Young, 1989; Modood et al., 1997, 2006; Meer and Modood, 2016).

As they are closely linked to the British context of integration, liberal and multicultural and intercultural theories of citizenship will be the focus of this theoretical framework. Despite variations in the scope of rights, liberal theorists share a commitment to the universalist and individualist nature of rights, that is to say, similar rights to all citizens as individuals and not groups (Kymlicka and Norman, 1994; Bloemraad, 2000; Lister and Pia, 2008). Liberal rights are seen as difference-blind, where citizens, no matter what differentiates them, should be allocated universal citizenship status before the law (Barry, 2001). This has been perceived by several scholars as homogeneous, assimilative, essentialist, yet exclusive of the other who differs from the national identity (Young, 1989; Walzer, 1994).

Arguing that state neutrality is in practice ‘hypocritical’ and often ‘incomplete’ (1994, 102), Walzer indicates that most liberal states do not exhibit neutrality when it relates to the majority culture. For the sake of securing the cultural survival of the state, unneutral public recognition and support are always offered to things such as ‘the language, history, literature, calendar, or even the minor mores of the majority’ (Walzer, 1994, 100). Citizenship is a promise of equal status and recognition similar to that obtained by ‘native’ citizens. Yet, the ‘legal’ inclusivity of liberal citizenship is not necessarily mirrored in practice (De Genova, 2015). Drawing a line between acts and the practices of citizenship suggests that citizenship ‘goes well beyond legal ascriptions of nationality’ (Lewicki and O’Toole, 2017, 153). The existence of official rights *per se* are not sufficient to provide equal citizenship status for non-majority groups (McGhee, 2008; Anderson and Hughes, 2015; De Genova, 2015).

Multiculturalists on one side (Young, 1989; Modood et al., 1997, 2006; Meer and Modood, 2016) and interculturalists on the other (Cantle, 2012, 2016; Zapata-Barrero, 2016, 2017) emerged in response to contemporary cultural diversity. Criticising the liberal uniform understanding of rights for failing to account for the complexities of diverse societies, the notions of ‘differentiated citizenship’, ‘inclusive citizenship’, and/or ‘multicultural citizenship’ began to dominate citizenship debates during the 1990s for the sake of group representation and recognition of minority cultures (Young, 1989; Taylor, 1992; Kymlicka and Norman, 1994; Joppke, 1996; Lister 2007; Meer and Modood, 2016; Meer et al., 2016). Interculturalism, in an attempt to incorporate ethnic minorities (Muslims in particular) into the national mainstream, has been founded on fears and anxieties over national fragmentation. Inherent in the intercultural turn is a portrayal of Muslim identities as deviant from what is desirable and what is understood to be normal in British society (Ryan, 2011; Khan, 2014).

The stereotypical construction of Muslim communities has played a role in stigmatising these communities as a source of fear for the wider society. Associating Islam with terror, violence, and extremism has played a crucial role in spreading a deep and sustained fear of Muslims in majority populations (Peucker and Akbarzadeh, 2014, 81–82). In intercultural studies, the concept of fear is often used to describe the worries of native populations over increasing diversity, which is seen as a threat to the cultural purity of the society (Home Office, 2001; Foner and Simon, 2015; Cantle, 2016). Rising fears and anxieties of cultural fragmentation have consequently contributed to the shifts in citizenship theory. Whereas multicultural citizenship is mainly concerned with group-based identity rights, intercultural citizenship is concerned with individual-based identity rights, along with national identity. Interculturalism is built on the ‘threat hypothesis’, where minority identities are perceived as a threat to national unity and social solidarity (Bouchard, 2011).

Increasing social and economic inequalities, ignoring the worries of majority populations, and sowing the seeds of so-called ‘Islamic’ terrorism are examples of what multiculturalism has been linked to in Britain (Joppke, 2009; Cameron, 2011; Cantle, 2012; Maxwell et al., 2012; Cantle, 2016). Interculturalism comes as part of an anti-multicultural debate where ‘cultural excess’ and recognition of ‘illiberal’ cultures are perceived as social problems (Zapata-Barrero, 2017, 5). Mapping onto UK politics, urging Muslims to identify with mainstream culture while departing from their traditional un-British values has been argued as a way to secure belonging for rootles Muslim generations (Cameron, 2011; Casey, 2016). Debates on Muslims’ lack of belongingness therefore call for greater understanding of their experiences and feelings of Britian as home. In this context, this study sheds light on the experiences of Muslims who identify as British but are *forced* into feeling as though they are exiled by wider socio-political narratives.

## Methodology

This analysis is informed by a 12-months of ethnographic research in Balsall Heath, a Muslim concentrated neighbourhood in the city of Birmingham, seeking a detailed account of relevant meanings and experiences of integration among local Muslims. I conducted 29 semi-structured interviews with Muslims from different generational cohorts, age groups, ethnicities, and religious orientations in order to develop an in-depth understanding of a problematised community which is often perceived as non-integrated (Casey, 2016). Alongside the interviews, locals’ behaviours, activities, and relationships were closely observed with the aim of gaining additional interpretations of insiders’ perspectives. These field observations and the 29 interviews aimed to explore the way Muslims identified themselves and expressed their attachments and sense of belonging in the United Kingdom.

It was not only for practical reasons (securing access) that I chose to carry out my fieldwork in Birmingham, but also for considerations such as its long history of immigrant settlement and long-standing diversity (Rex and Moore, 1967; Solomos and Back, 1995; Spencer, 2002). The city has one of the largest Muslim populations in Britain and a range of Islamic sects and schools of thought, accounting for about 21.8% of the city’s population according to the 2011 census (Birmingham City Council, 2018; Knott, 2018). Of particular relevance to the focus of this study, Birmingham is one of the main cities described as being highly segregated (Casey, 2016). In terms of Muslim residential patterns, the city includes wards with more than 70% Muslim populations, a matter which is highlighted as alarming in government-sponsored research and official integration accounts (Cameron, 2015; Casey, 2016, 11).

My participants included only Muslims who are citizens and/or permanent residents, with the aim of speaking with those who had a better understanding of British society, culture and laws, and who were expected to have a clearer understanding of their rights and duties compared to short-term residents. Variations in the sample were considered to include different ethnicities, age groups, genders, careers, religious orientations, and level of educational qualification to achieve a representative sample of British Muslims. All interviewees were British citizens except three, and these possessed permanent settlement status. Interviewees were all of immigrant backgrounds: Pakistani, Yemeni, Moroccan, Malaysian, Saudi, Afghani, Egyptian, Sudanese, Afro-Caribbean and Lithuanian. The sample included 15 females and 14 males; 13 were first generation immigrants, 12 were second generation and four were third generation. All participants were in current employment except for nine participants, of which six were homemakers, two were university students, and one a pensioner.

In terms of age, the sample included three groups, the first was made up of 18 participants who were at the time of interview between 20 and 39 years old; the second was made up of nine participants who were between 40 and 59 years old, and the third group included two participants who were 60 years old or more at the time of interview. In terms of education, eight participants held master’s degrees, 11 held bachelor’s degrees, eight held GCSE (or lower) qualifications, and two were university students. In the context of this article, all participants’ names have been replaced with pseudonyms.

In line with my ontological stance, this study is data-oriented rather than theory-oriented. Following this approach entails that data occupy a central position compared to theory, unlike positivist approaches to research, in which the research is tested against existing theory (Van Maanen, 2011). As an ethnographic research project, this study is committed to ‘analytic induction’, where the aim is to build generalisations and inferences from insiders’ perspectives, behaviours, acts, activities, and relationships (Brewer, 2000, 108–109). During my fieldwork, I was committed to revealing the meanings British Muslims assign to the self, the other, the space and the place, social relations, diversity, and the surrounding context. Understanding these meanings was key to answering my research question of how British Muslims understand integration as a multidimensional concept comprised of individual, social, and contextual aspects.

The research project was set out to engage with debates over Muslims self-segregation, ghettoization, and lack of integration in general. Despite being data-oriented, it was, however, important to consult relevant theories and previous scholarship to structure my interview outline (Vidich and Lyman, 2000). This study adopts an open definition of integration to suit the rationale of field investigation. The open definition of Phillimore allowed me greater capacity to explore the community under observation without a pre-defined sense of the meaning (or purpose) of integration. As Phillimore states, integration ‘implies the development of a sense of belonging in the host community, with some renegotiation of identity…’ (Phillimore, 2012, 3). Based on this definition, I aimed to explore British Muslims’ sense of belonging and the way they perceived and experienced it: What contributes to the development of their sense of belonging and what might hinder it? Belonging is an emotional attachment that relates not only to feeling ‘at home’ but being confident about the right to share the home (Yuval-Davis et al., 2018, 230). It is grounded in ‘a felt connection to self, others, and place’ (Pearlman, 2023, 165). The literature reveals that political, economic, social, and cultural factors influence the sense of belonging and the process of integration (Castles et al., 2002; Ager and Strang, 2008). Interview questions were therefore designed to elicit details on a range of questions that closely link to the concept of integration, such as citizenship, rights, political participation, social mobility, employment, housing, belonging, intergroup and intra-group relations, and barriers to cohesive community relations (Ager and Strang, 2008).

The second research technique, participant observation, was used to uncover how integration practices and behaviours were maintained during normal day-to-day interactions. On a daily basis I was directly or indirectly observing human affairs and interactions to better understand the community’s actual behaviours in terms of integration, and to compare this with what I gleaned from my interviews and casual conversations with the people (Fetterman, 1998). In the field setting, I attempted to be both participant and observer. As participant, I joined in attending workshops, conferences, coffee mornings, and meetings in local organisations and with community groups, and as observer, I made observations during my daily routine activities, such as going to markets, shops, schools, and/or parks where casual and informal conversations enhanced my understanding of the context and the experiences I witnessed.

## Analysis: navigating exilic space

The findings uncover the complexity of integration as a process that constructs a range of paradoxical feelings and experiences for Muslims in the United Kingdom. Engaging with two major themes of the findings, the analysis is divided into two main sections. The first section discusses -in its two subsections- how both fear and citizenship inequalities construct a space that is exilic in nature hindering possibilities of belonging. The second section explores how British Muslims react to exilic narratives through their approaches to maximise their sense of being ‘at home’.

Despite unhesitatingly identifying as British, these Muslims, including British-born generations are often forced to negotiate conflicting circumstances and conditions that largely outcast them and put them at a distance, where they are unable to belong. Even when they feel that they belong to the United Kingdom, they often, to varying degrees, get bounced back by wider exilic narratives and conditions to their ethnic/religious boundaries. My participants had to adapt to live in what I call an ‘exilic space’—one that restricts their ability to develop an unconditional sense of belonging. In one way or another, most of my participants were touched by the realities attached to this exilic space, grounded largely on both fear and an incomplete sense of equal citizenship. This section will be therefore divided into two subsections: the first to discuss the role of fear and the second to discuss the inbuilt inequalities of citizenship.

### The role of fear

In observing Muslims going about their normal daily activities one may not be aware of the existence of fear within the community. During the early stages of my fieldwork, no signs of fear among Muslims were clear to me. However, as time passed, it became clear that in fact there was much unknown behind what I initially perceived as a normal life. Muslims’ lives were clearly not as smooth as they seemed to be. For many in the field, fear was present in daily life, controlling not only what they did but also where and when. This was verbally articulated during interviews and in the course of my interactions with the community, and it was reflected in their behaviours and choices, for instance, restricting their residential choices and identity display. This fear was demonstrated during the interview recruitment process, where many potential participants declined my interview requests, repeatedly expressing that they ‘do not want to get into trouble’. Many in the field anticipated negative consequences as a result of participation in my research, given the ongoing racialisation of the Muslim community, getting involved in research related to Muslims could be a potential source of danger if their narratives were misinterpreted or taken out of context.

The level of fear amongst skilled and/or socially mobile Muslims was relatively lower compared to those less privileged in these regards. However, it cannot be overlooked that these fears existed across the community and within all its ethnic groups, and across educational attainment levels, socio-economic status, and generations. Aminah, 25-year-old third-generation, was a non-hijab-wearing woman with a university degree and a running successful business. She expressed her fear of what might happen to either of us as a result of such a discussion/interview. She said, ‘If they [the authorities] get to know about this, we will be in a big trouble.’ I followed her comment by asking, ‘Why? I am only a student doing her doctoral research work. I have all the proof, so do not worry.’ She replied, ‘You know what’s going on.’ Aminah’s account relates not only to issues of fear and mistrust of how authorities might deal with her being a Muslim, but also refers to an exilic context which involves exclusionary and stereotyping reminders of Muslims as ‘other’.

My participants’ fear over how they were perceived and interpreted was associated with their sense and awareness of being part of a stigmatised community that is ‘constantly surveilled, scrutinised and silenced’ (Acik and Pilkington, 2018, 152). It was because of this ontological insecurity that my participants spent a great deal of time and effort ensuring that I was not misunderstanding or misinterpreting them or their words, as they felt a simple misunderstanding on my part could mean a lot of trouble for them. This context rendered many participants fearful of speaking up, voicing their disagreements or showing their difference in a context where, for example, criticising policy agendas such as Prevent, brands dissenters not only as non-integrated but also as being on the side of extremists (Spalek, 2010; Choudhury and Fenwick, 2011; Hymas, 2018; Kapoor and Narkowicz, 2019). In this context, Choudhury and Fenwick argue that compared to any other community, British Muslims are more likely to ‘believe that the police will treat them worse than people from other racial groups’ (Choudhury and Fenwick, 2011, 15). That said, I came to understand the fearfulness and mistrust voiced by my participants, such as the second-generation 48-year-old Ijaz when he stated that:

People are afraid that things will get taken out of context. The Muslim community does feel under scrutiny now. They do feel under attack. The mistrust, I think, comes from projects like Prevent…there is a lot of mistrust in communities about Prevent.

The exilic discourse of integration was also a source of fear for my participants. Many were fearful of being labelled as non-integrated, and therefore often highlighted how they fit into and participated in the broader society as every other community did. At the site of my fieldwork there was indeed no single Muslim community, and no single voice to explain why Muslims may opt to integrate or not. However, for most of my participants, integration is a politicised concept which is instrumentally used to exclude those who might not agree with the mainstream understanding of what being British means. It is also perceived as exclusionary, as it aims to integrate those whose difference is undesired and cannot be tolerated. In this regard, contemporary integration discourse is indeed a threat to a well-rooted sense of belonging. Uzmah, a second-generation 47-year-old woman shared this point of view, saying:

When we say integration, I think again that is categorising, and we subconsciously categorise everything. Okay, to me integration is having no boundaries between people, no labels, so we are a community as a whole. Like when we were growing up we never looked at the fact that our neighbours were black or white or Indian or Chinese. They were our neighbours. And we have created labels which now have segregated us; and this issue of integration has become really an issue.

My participants found that this implicit/explicit labelling is divisive and does not help facilitate social inclusion for minorities in general and Muslims in particular. Being singled out in integration debates was overwhelmingly seen as undermining to Muslims’ position as equal citizens. Answering my questions ‘What role should Muslims play to narrow the social distances between them and the British communities?’ and ‘Do they have any responsibility in enhancing integration?’, Uzmah carried on, saying:

I do not think we have to be taking any special responsibility because we are British and we are Muslim. I do not think so. *I think we all need to take the responsibility because we are humans and citizens*, you know. If we all take our responsibility as citizens then I think that is when the change begins to happen. If we start to look at it that, because we are Muslim, we need to behave in this way or we have to make an impact or we have to…

That need for Uzmah, and nearly all my participants, to emphasise common sense entitlements such as her position as a citizen (let alone a human) brings to the fore a sense of unsettled belonging, but most seriously it signifies a cluster of paradoxes of both belonging and exclusion that are normally constructed in exiles. Putting the responsibility for segregation on the ‘alien others’ against the ‘homogeneous tolerant’ society is a discriminatory bias that works against the expected outcomes of integration (Blommaert and Verschueren, 1998). An equal representation of all communities in the integration/segregation discourse reflects the soundness of equal citizenship in a particular country. Criticism of this one-sided focus of the integration discourse was significantly echoed amongst my interviewees. For instance, Sarmad, a second-generation 45-year-old man stated: ‘It is true that a considerable portion of British Muslims prefer to stay near their families, however, it is equally true that the white community has left these Muslim-concentrated areas’. Sarmad continued, ‘I think people choose to live here because their mothers and fathers and their brothers and sisters live here. They do not want to go further away. You should be interviewing the non-Asian people about why they are moving out of these areas.’

Fear of being labelled as ‘non-integrated’ was also accompanied by fear of integrating according to restrictive assimilatory policies seen by many as a threat to diversity and identity-related gains (including recognition of difference). The vagueness of integration as a concept and an agenda intensified my participants’ fears over the thresholds and boundaries of integration policies and whether or not these polices undermine their already earned citizenship and diversity privileges, particularly in regard to freedom of difference. Criticising the way recent integration debates are framed, Adam, a first-generation 44-year-old, said:

If people understand integration as a replacement of foreign [non-mainstream] identities, that is a wrong understanding of integration; and that is worrying. That is not integration. Integration means cooperation, doing your duties and respecting rights, respecting the law, helping the community; being a helpful part in the community; being loyal to this country. This is integration, and that is not to do with my feelings with my identity or my religious thoughts.

As a first-generation immigrant, Adam articulated that coming to Britian was for him (and his family) a cherished opportunity to be part of a land where he dreamed of agency and having his voice heard. The quote from him above refers to this balance between being shaped by the world and being able to shape the world, something which not only develops the necessary sense of a stake in the integration debate but most importantly emphasises feelings of belonging and the sense of being ‘at home’. As Jackson argues, ‘we often feel at home in the world when what we do has some effect and what we say carries some weight’ (Jackson, 1995, 123). Exiles are normally both restrained from doing and coerced to do/act in certain ways that are most likely forced upon them. They lack agency and hence lose that sense of stake in issues that matter to them. It is no exaggeration to argue that the position of Muslims in integration debates has a range of resemblances with how exiles normally feel. Muslims’ unheard voices in the integration process and their exposure to otherness and misrecognition signify exile-like conditions, but without—in the case of British-born generations—geographical displacement. Their day-to-day experiences of fear in all the forms discussed above are representative of a marginal existence in relation to public and political mainstream discourses.

### The myth of equal citizenship

A sense of home and the ability to integrate was strongly associated, by nearly all my participants, with a sense of equal citizenship. Equality for my participants was the central pillar to their understanding of integration and a prerequisite for feeling ‘at home’. Ijaz, 48-year-old second-generation, emphasised the role of equality in promoting integration and enhancing sense of belonging for Muslims, stating:

I think what really encourages integration is when everyone feels equal to one another. I think that is the catalyst. When each citizen feels equal to the other citizen. When you have a feeling of inequality, like one group feels that they are being treated less than the other group or they do not have the same opportunities, here you get a feeling of inequality and here you start getting conflicts. Wherever you see conflict in the world there is always one group who feels that they are being treated less, or being given less opportunities than the other group. And that creates conflict. So, I think the foundation of integration has to be a feeling of equality where all groups feel equal to one another. The people who came in the 1950s and 1960s, I don’t think they did feel that equality. I think they were given the worst jobs, and they were treated unfairly. And they did not also know the language. So, because of that feeling of inequality, they did not really integrate. The big rock of integration is the feeling of equality, and the feeling of being on the same level as your brothers and sisters of white communities, or of Afro-Caribbean communities and so on. Then you can feel part of that society.

For decades, multicultural citizenship in the United Kingdom was understood to mean recognition of difference and equality, and as a way to tackle racism and discrimination. However, recognition of difference may work rather as a form of othering and marginalisation. Measures to protect equality could become just symbolic ‘tick box’ rather than promoting a real and genuine feeling of equal citizenship. In response to a question on whether she feels equal to her white British peers, Iman, a 29-year-old woman of Arab-English background said:

No, I think sometimes I have to work harder. Sometimes you have to work harder and you might get the opportunity to just fill a box not because you will add something, but because they need to fill some quota.

Similarly, the 23-year-old third-generation Ayman who strongly valued his religious identity and Islam as a way of life, defended his right (and that of every other community) to have his own beliefs. At the same time, he called for not turning that respect for difference and the right of religious and ethnic minorities to practise and preserve their identities into othering, saying:

We need to stop saying white people, black people. Just people. Stop saying Muslims… Pakistanis. Just people. Citizens. Once this becomes universal and nobody tells you who you are ethnic-wise and nobody mentions you by a certain label, then that is it, everyone is sorted, no problem.

In the field setting, there was agreement amongst my participants that preserving cultural differences should not shake the fundamental principle of citizenship, which is to treat citizens as *equals*. Minority rights do not have to work as stigmatic markers of minority groups as un-equals. That is, treating ethnic minorities as equals entails not only securing their right to the preservation of their cultural identity, but also protecting them from being seen as unequal (Siim and Squires, 2014). Being singled out in integration discourses was seen by the majority of my participants as an obvious for of unequal citizenship; and therefore integration for many in the field was identified first and foremost as ‘equality’. Integration is a conditional process, which should be founded on values of equality, mutual respect and freedom of difference. The central position of equality and freedom of difference in Muslims’ understanding of integration was demonstrated by Hamza, first generation 43-year-old, when he said:

When you ask about integration of Muslims, you should look first for how to assess their integration. What you mean by integration? Do you want me to dissolve in your culture so that I become integrated? Or, do you mean that I give the best I can for this society, and you also give the best you can for this society? Do not use the integration card. If you look at the indicators of Muslims’ numbers in politics, medicine, science, engineering, you will see Muslims are integrated. This word can be used in many wrong ways. It can be like: if you do not follow my lifestyle, you are not integrated. If you do not follow me, you are not integrated. If you do not eat what I eat, then you are not integrated. Integration does not mean that I lose my right to practice my identity. If I do not dissolve in your culture, this does not mean that I am in contestation with you. I should have the right to identify myself without fear. I am Jewish, I am Muslim, or I am Christian. I do not have to dissolve in what politicians want to be named integrated. So, I prefer the concept of equality of rights instead of integration. You respect my rights and I respect your rights.

My participants’ sense of belonging seemed, however, to change according to the context and the situation. A lowered sense of home and hence an amplified sense of exile was more likely to be present at times where citizenship rights are at stake and are questioned (e.g. times of fear, discrimination and/or racism). One of my participants, a third-generation 25-year-old woman, narrated that her experience at school in the time following the London bombings (2005) was a turning point not only in her relationship with her faith but also in her perceptions of her Britishness. Recalling her experience in secondary school following the 7/7 terrorist attacks, she said:

It was just after the London bombings that happened in 2005 that things suddenly changed. It was like everything was fine until that happened. For some reason we were then targeted. People were asking us questions that we did not have answers to but I was in a different place, I was young. It is *those* Muslims [the bombers] or whatever they claim to be. It is not *us*. For that we have no responsibility. We do not have responsibility for what they did. I knew it was a change happening at my sixth form at that time. [… ] That was the first time in my life ever that I noticed what being British was or what it was being Asian British.

My participants often made clear their awareness that being recognised as British was conditional upon being ‘good citizens’. Their awareness of this rendered them feeling discriminated against and disowned by British integration politics. Recalling her experience in secondary school after the 2005 London bombings, the above-mentioned participant followed up, saying that ‘from that point, a lot of things have changed in schools’. Being targeted or suspected initiated in her a feeling of being lesser compared to a white fellow citizen, and it also divided society in her perspective along lines of religion and ethnicity. Wider narratives on Muslim self-segregation and rising anti-Muslim sentiments from the 2000s meant that many British-born Muslims—and first-generation Muslim immigrants too—were let down into an exilic space where they have felt suspected, targeted, and alienated. She explained this when she said:

Imagine living in a time when constantly you have to prove yourself. You go to work, you have to speak to everyone, you have to prove yourself that you are a good Muslim. You go outside, you have to prove that you are a good Muslim. It is becoming a headache, you know: you constantly have to prove. When people are asking you questions and you do not have the answer you do get confused. So, you know, okay, I am still a Muslim but I need to be able to defend myself. So, I felt like that was the first time in my life ever that I noticed what being British was or what it was being Asian British-- what all these different communities to white British are like.

British Muslims’ fears over social acceptance relates to notions of Britishness and belonging. A limited framework and definition of Britishness and who is considered British allows more room for the exclusion and isolation of many who carry immigrant backgrounds. Racialising identity and religion is a significant barrier to achieving full citizenship in law and in practice. As Sharma argues, ‘Racist definitions of national belonging results not only in the refusal to admit certain non-citizens but also in the social refusal to accept many *co-citizens* in the political community’ (Sharma, 2015: 99). On empirical grounds, I argue that the United Kingdom has developed a differentiated form of citizenship that does not necessarily protect what I call ‘equality of belonging’, that is, the opportunity for every citizen to feel they belong and that this belonging cannot be denied based on his/her cultural or ethnic difference. Lacking recognition of belonging is a constitutive part of an exilic space, where Muslim minorities struggle with otherness and marginalisation.

### Constructions of belonging in exilic context

Negotiating the above exilic narratives and experiences, my participants’ responses varied between fight or flight depending on age, generation, gender, and socio-economic status. The nature of the response was based on the ability of the person to take either course of action in the context of social and personal circumstances. With wider anti-Muslim sentiments, some participants chose to adhere to their positions as ‘others’. This was reflected in some of my participants’ choice of place of residence. Areas with co-ethnic links consolidated their sense of security and worked as a collective counterpower against exilic experiences such as fear of racism. Perceived/actual racism and/or prejudice increased their psychological and emotional need for close ethnic links to promote their sense of safety. Ethnic togetherness decreased my participants’ sense of vulnerability when they anticipated/faced racism and needed easier and safer conditions in which to practise their ethno-religious identities. Hania, one of my second-generation participants, linked her sense of comfort living in Balsall Heath with her anticipation of social rejection, racial harassment and racism should she live outside the area. She said:

I have lived in Balsall Heath my whole life. I never moved out of the area. I stayed within my comfort zone…I am worried if I move to a different area that probably I will get errrm, like in some areas. Like when I used to go to school, I used to get harassed on the bus by English people…it was a really racist area. When I used to go to school we used to get harassed by big boys and women.

It was common to hear positive comments from my respondents about how they valued acknowledgement of their religious difference in the United Kingdom. Nonetheless, this optimistic outlook does not deny the difficulties many Muslims experience impacting on how they actually think of and experience their religious identity and thereby their sense of belonging. Sarmad, second-generation 45-year-old, was mostly liberal in his narratives and in favour of intergroup contact and mixedness. Yet, despite his liberal views and his privileged socio-economic circumstances, Sarmad’s anticipation of racial assault shaped his movements within the city of Birmingham. As he said:

If I wander into a very kind of council-white area, I will get probably-- some kids or whatever-- abusing, shouting or something. But you just kind of avoid those areas. I do not have reason to be in those areas so I do not go. There are probably pockets of areas within the UK and Birmingham possibly that you will experience it [racism]. And maybe that is because they are not used to seeing Asian faces.

In my field setting it was strongly noticeable that ethnic-concentrated areas are places of social relief for many Muslims (and for immigrants in general). In such areas, Muslims/immigrants face fewer social pressures from integration/segregation discourses. My participants often expressed that in dominantly Muslim areas they were not obligated to provide explanations for their religious behaviours, ways of dress, or lifestyles. This search for mutual understanding and social acceptance answers the question of why many of my participants chose homes in neighbourhoods where they do not feel misunderstood because of their identity and need not explain or justify their choices to others. In Muslim-dense areas such as my field site, I observed and experienced that sense of freedom in expressing the self which enhances one’s feeling of being at home. I could see that in these areas no one is held accountable for how integrated/non-integrated he/she is in terms of dress, religious practices, beliefs, and/or behaviours. Despite the number of socio-economic challenges, from my own experiences and from my social interactions in the field, I often found that ethnic/Muslim areas incubated and promoted a sense of home for its residents which they might not be able to enjoy in non-immigrant areas. Ethnic proximity represented a refuge for many Muslims against problems of social acceptance and social isolation. Their togetherness, consequently, enhanced their wellbeing and sense of community and increased their sense of belonging.

For some, the negative impacts of exilic narratives extended to non-spatial aspects of their daily lives, affecting behaviours and attitudes towards integration in the wider society. The alienating impact of living in a state of exile made many at ease with a withdrawal from public issues that do not closely relate to them. For such participants, the condition of exile is a manageable space at some times, and a comfort zone at other times. Not everyone felt this way, though. I came across some Muslims who opted not to stay silent but to resist wider exilic narratives by crossing out of their comfort zones. While the response to a felt state of exile for some meant withdrawal and opting for safety and maintaining the status quo, it was seen as cause for confrontation and change for others. This resistance was displayed by some participants through identity-related reactions. Longing for a sense of belonging, some participants responded to the exclusionary exilic conditions they endured by increased religious assertiveness. For the above-mentioned Aminah, religious assertiveness was a reaction to the stigma that followed the 7/7 bombings. She said:

I am quite a strong headed person so to me it was like ‘I need to answer their questions’. I would go to the internet, researching and reading on Islam to know what Islam says about these things. I actually became closer to my religion than ever before. It took the opposite effect for me because when people are asking you questions and you do not have the answer you get confused, so, you want to know more. I am still a Muslim but I need to be able to defend myself.

Such a form of reactionary identity formation lessens the social pressure of stigma and discrimination and plays an emancipatory role against the overall exilic environment because, not only is it a form of resistance, but it also engenders a sense of belonging and empowerment. The ability to ‘exclude the excluder’ provides the vulnerable among my participants with a sense of agency over a dominant sense of powerlessness and victimisation (Castells, 2010, 9). Affluent participants, those with higher socio-economic status, and those with extended family and ethnic ties—were more likely to maintain their sense of belonging through active resistance to exilic experiences such as stigma and prejudice. This enhanced social status made these participants feel more able to express dissent with the exilic discourses. For the majority, however, their challenging socio-economic conditions forced them to stay silent to avoid adding extra complications to their lives.

A significant proportion of my participants, particularly younger generations, employed what I call ‘identity tailoring’ as a commonly followed approach to gain recognition in the wider society through making adjustments for their Muslim identity. They adopted pragmatic and non-confrontational approaches regarding their religious practices to achieve a form of cultural reconciliation that helped them overcome their exilic experiences. For instance, many young Muslims presented their inherited religious identity as a symbolic affiliation, a compromise which they expected would secure them a position between the two cultures.

While for some this was a personal choice and a reflection of their understanding of what a Muslim religious practice involves or should involve, for others it was a conscious—though hard—decision for the sake of gaining recognition and securing a sense of belongingness. Taking this harder route with all its implicit/explicit exilic connotations, this group would identify themselves as Muslim but would not necessarily commit to religious-based views and/or choices. Obvious Muslimness (such as wearing a head covering) would add to the complexities a Muslim might experience when interacting in their environment. Here, I recall a meeting with Carrie, a first generation 33-year-old Estonian white convert, who confirmed that she wore the hijab only within the confines of predominantly Muslim populated neighbourhoods because she did not feel safe enough to do so elsewhere. Facing this challenge, Carrie expressed her difficulty feeling home in and belonging to the United Kingdom.

While finding that she needs to ‘work harder’ being a British Muslim, the above-mentioned Iman, 29-year-old English-Moroccan, reflected on the complexities of identity display in a context where her face-veiled mother would not visit her home, located in a predominantly white area, fearing religious-based harassment. Other participants explained how they were discouraged from, for example, sending their children to Muslim schools and/or overtly exposing their religious identity because this might impact on their later on opportunities in the labour market. Those participants felt coerced by a context of prejudice and stigma to limit their involvement with and practice of the faith in order to secure a more stable position in the wider society. Downplaying their religious difference and compromising certain Islamic rituals (e.g. prayers and headscarf in the workplace), this group felt more secure about gaining social acceptance. Though these identity adjustments represented for some a route to fitting in, it was simultaneously a representation of otherness and forced in-betweenness that hampers connectivity to the authentic self.

There was, however, another group of participants who were not able to position themselves amid paradoxical exilic experiences. Nearly all my participants found the above paradoxical context problematic in terms of their sense of belonging. Young participants, however, were more likely to express their struggle with a sense of homelessness, feeling that they are recognised neither in their parents’ homeland nor in the United Kingdom. This confusion was expressed by the third-generation 22-year-old Sabrina:

When we go to Pakistan, they are like, ‘they are British’, ‘they are not really Pakistani’, ‘they are British’. So, where do we fit? The British say we are not British, and Pakistanis are saying we are not Pakistani. Well, who are we?!

This quote unmasks the feelings of exile and estrangement despite coming from a person of two seemingly rooted identities. In the case of young British Muslims, the fading link to their parents’ ethnic and religious communities is simultaneously paralleled with a weak sense of inclusion in the wider national community. In this context, they are likely to be left in a vacuum with a doubled sense of alienation. Failure to achieve personal self-fulfilment may lead to a state of being ‘culturally homeless’ (Tucker, 1994, 183). As Zeng puts it, being in a ‘vagabond’ state and longing for a lost centre—both signify the exilic experience making up a state where one feels estranged and unable to belong (Zeng, 2010, 2). This condition meant that young Muslims were likely left with no direction, no purpose in life, no ability to contribute to society, no sense of significance, and no points of reference. In other words, it left many feeling *homeless* despite being home.

## Conclusion

This paper provides a response to decades of heightened public and political debate demonising Muslim communities for their lack of integration into British society. Engaging with the accounts of integration amongst British Muslims, this paper argues that Muslims in the United Kingdom endure a challenging context where barriers to integration leave them not only unable to integrate but, more seriously, not feeling home in their homeland. Ethnographic investigation has unearthed the reality that Muslims in the United Kingdom may end up being locked into an *exilic space*—one which renders them entangled in fear, inequality, exclusion, and a reduced ability to belong. In a state of belonging, one is able to maintain a connection to the self, others, and place to the fullest. For my participants, there are, even without mentioning the word ‘exile’, stories that relate to meanings of exile—ones which are paradoxical and involve feelings of being insiders and yet kept at a distance like outsiders at the same time. Muslims’ stories and daily experiences often conveyed a sense of both home and exile, in overlapping and varying levels. However, generally speaking, my findings suggest that a sense of home is an outcome of a complex of parameters that work together to create a citizen ‘able’ to feel at home and able to belong. The ability and willingness of British Muslims (and ethnic communities in general) to integrate into and to take part in society is largely shaped by a set of wider variables that either stimulate or impede both their will and ability.

Muslims in my field site identified themselves as British and identified Britain as home. Younger Muslims were less vulnerable in terms of social isolation compared to their first generation ascendents. Yet, with the rise of anti-Muslim narratives and sentiments, they had a sense of exile forced upon them. They are conscious that as Muslims they need to work harder to demonstrate their belonging—a recurring experience that reinforces the shades of exile in a country they consider home. Exile was and still is a constant experience for British Muslim communities, with both their foreign-born and British-born generations. While first-generation Muslims may undeniably endure a sense of exile as they long for the homeland while living in the diaspora, it is particularly important to question the experiences of British-born Muslims who, to varying extents, endure exilic conditions that make them categorically feel not at home in their own homeland. For home-born generations of Muslims it is a ‘metaphorical’ exile into which they are forced because of social othering and exclusion, unlike their first-generation ancestors, whose sense of exile was a response to a geographical displacement.

At my field site, many were not distant but distanced, not silent but silenced, and not segregated but exiled. By engaging with those who undergo exilic conditions—whose voices may go unvoiced and unheard—this paper concludes that unacknowledged integration struggles and frustrations turn out to be not only alienating but exilic in nature. Yet despite the challenging cues, these Muslims show a strong ability to negotiate their national and religious belongingness and to preserve their sense of Britain as home. British Muslims have come to terms with their belonging, but it has been perceived largely through the lens of ‘exile’. Exilic conditions, however, do not denote a state of utter homelessness. Where these exilic conditions existed and felt, it was likely to lead to a stronger inclination towards segregation. Segregation in this case was instigated by feelings of uncertainty, fear, inequality, and alienation—that together created feelings of being *exiled at home*.

## Data availability statement

The original contributions presented in the study are included in the article/supplementary material, further inquiries can be directed to the author.

## Ethics statement

Fieldwork used in this article was approved by Keele University Ethics Committee. The studies were conducted in accordance with the local legislation and institutional requirements. The participants provided their written informed consent to participate in this study.

## Author contributions

SE has conducted the ethnographic field work, analysed the data, and written this paper.
